# The Maternally Expressed WRKY Transcription Factor *TTG2* Controls Lethality in Interploidy Crosses of *Arabidopsis*

**DOI:** 10.1371/journal.pbio.0060308

**Published:** 2008-12-09

**Authors:** Brian P Dilkes, Melissa Spielman, Renate Weizbauer, Brian Watson, Diana Burkart-Waco, Rod J Scott, Luca Comai

**Affiliations:** 1 Section of Plant Biology and Genome Center, University of California Davis, Davis, California, United States of America; 2 Department of Biology, University of Washington, Seattle, Washington, United States of America; 3 Department of Biology and Biochemistry, University of Bath, Bath, United Kingdom; Max Planck Institute for Developmental Biology, Germany

## Abstract

The molecular mechanisms underlying lethality of F1 hybrids between diverged parents are one target of speciation research. Crosses between diploid and tetraploid individuals of the same genotype can result in F1 lethality, and this dosage-sensitive incompatibility plays a role in polyploid speciation. We have identified variation in F1 lethality in interploidy crosses of Arabidopsis thaliana and determined the genetic architecture of the maternally expressed variation via QTL mapping. A single large-effect QTL, *DR. STRANGELOVE 1* (*DSL1*), was identified as well as two QTL with epistatic relationships to *DSL1*. *DSL1* affects the rate of postzygotic lethality via expression in the maternal sporophyte. Fine mapping placed *DSL1* in an interval encoding the maternal effect transcription factor *TTG2*. Maternal parents carrying loss-of-function mutations in *TTG2* suppressed the F1 lethality caused by paternal excess interploidy crosses. The frequency of cellularization in the endosperm was similarly affected by both natural variation and *ttg2* loss-of-function mutants. The simple genetic basis of the natural variation and effects of single-gene mutations suggests that F1 lethality in polyploids could evolve rapidly. Furthermore, the role of the sporophytically active *TTG2* gene in interploidy crosses indicates that the developmental programming of the mother regulates the viability of interploidy hybrid offspring.

## Author Summary

Many flowering plants tolerate changes in the number of genome copies (ploidy), but offspring of parents with different ploidies often fail to develop. We investigated this phenomenon in Arabidopsis thaliana and discovered variation in the ability to survive interploidy matings. Two common strains (known as accessions), Ler and Col, are respectively permissive and intolerant when diploid females are mated to tetraploid males of Col. We mapped the genes responsible for this response and identified a major locus, which we call *DR. STRANGELOVE1*, on chromosome 2, and after finer mapping, defined the locus *TRANSPARENT TESTA GLABRA2* (*TTG2*) as the candidate gene. *TTG2* regulates the fate of interploidy crosses, and knock-outs of *TTG2* improved the outcome of interploidy matings in both Ler and Col. Furthermore, the two accessions differed in genotype and in expression of this gene. *TTG2* is an epidermal regulator whose activity affects seed endosperm development. Importantly, *TTG2* acts within tissue of the seed-plant, indicating that a transgenerational interaction is responsible for controlling the outcome of interploidy mating.

## Abstract 3

Hybrid lethality in crosses between diploids and tetraploids, plants with whole genome duplication, is determined by an epidermal regulator expressed in the maternal tissue that envelops the seed.

## Introduction

Postzygotic lethality of the hybrid offspring from crosses between divergent parents functions as a barrier that drives or reinforces speciation [1–16]. The molecular mechanisms underlying this response, however, are not understood. In plants, an example of F1 lethality occurs when progeny from crosses between newly formed polyploid individuals and their diploid progenitors fail during seed development. This lethality arises in the absence of any allelic diversity due to the difference in the dosage of genomes provided by the parents. The strength of this reproductive isolation can vary within species and between individuals of the same species, and has long been known to contribute to speciation. Yet, we understand neither the genetic basis of this barrier nor the source of variation within and between species

Fertilizations between haploid and diploid gametes can occur in diploid species due to meiotic errors that produce unreduced (2n) gametes. In humans, for example, triploidy is associated with spontaneous miscarriage, causing postzygotic lethality in approximately 2% of all conceptions between parents of apparently normal karyotypes [17]. In diploid plants, the frequency of unreduced gametes can approach a few percent depending on the species, genotype, and environmental conditions [18–20]. Triploids from the union of normal and unreduced gametes can form tetraploid plants. Yet, the rates of new tetraploid formation are often orders of magnitude lower than unreduced gamete formation [9,21]. Still, the frequency of polyploid formation is greater than typical base substitution rates, such as estimated for *Arabidopsis* at approximately 1/10^8^ [22].

Postzygotic lethality in interploidy crosses is hypothesized to play an important role in polyploid speciation by genetically isolating populations of divergent ploidy [1–16]. Polyploid speciation is very common in the flowering plants, in which it is phylogenetically coincident with 4%–10% of all plant speciation events [21]. In addition to recent polyploid speciation events, whole-genome duplication from ancient polyploidy events is a feature detected in many plant, vertebrate, fungal, and protist genomes [23–39]. Thus, the establishment of polyploid lineages and polyploid speciation has played a role in the evolution of all multicellular eukaryotic lineages.

Complete reproductive isolation is not always achieved following a shift in ploidy, and the penetrance of the interploidy hybridization barrier varies within and between species [9,40,41]. In species without strong postzygotic lethality in interploidy crosses, polyploid derivatives can be formed via the fusion of a single unreduced gamete with a normal haploid gamete. When the resulting triploids are fertile, they can give rise to tetraploid and diploid offspring via the so-called triploid bridge [9]. In some plants, including Arabidopsis thaliana [42], very little postzygotic lethality has been observed in crosses between individuals of divergent ploidy, and the generation of tetraploids via a triploid bridge has been demonstrated [5,9,43,44]. Although this may increase the rate of polyploid formation, the recurrent formation of fertile triploids from interploidy crosses between tetraploid and diploid populations would permit the sharing of alleles between the two cytotypes and hinder polyploid speciation.

Work with karyotypic variants and interspecies hybrids by Nishiyama and Yabuno [45], Johnston et al. [16], and Lin [46] demonstrated that the contributions of the pollen and seed parents are unequal and that a balance between these contributions is required for normal sexual reproduction in plants. In interploidy hybrids, doubling the genome of only one parent upsets this balance and can result in seed lethality. Remarkably, species vary in the combining valence of their genomes such that some interspecies crosses are more successful when individuals of divergent ploidy are crossed. The term endosperm balance number (EBN) was proposed to describe the pattern of dosage-dependent incompatibility intrinsic to each species or accession [16]. Such phenomena are not limited to angiosperms. Vertebrate interspecies hybridizations, such as crosses within the genus *Hyla* [47], display increased fertility when the maternal parent is in genomic excess. The interploidy barrier is also affected by the direction of the cross, with maternal excess crosses, e.g., 4x × 2x, typically resulting in less postzygotic lethality than paternal excess (2x × 4x) crosses [9]. A similar effect is typically seen for postzygotic F1 lethality in plant species barriers, including the lethality found in crosses between A. thaliana and A. arenosa, in which increasing the relative maternal dosage increases seed survival [48,49].

The similarity of progeny phenotypes from interploidy and interspecies crosses, and the shared sensitivity to changes in genome dosage, has led a number of authors to hypothesize that both forms of F1 lethality could be controlled by the same mechanisms [16,49–52]. One expectation has been that variation in imprinted genes, in which expression in the offspring depends on the parental origin of the chromosome, is responsible for the postzygotic lethality observed in interploidy crosses [46,52–57]. As both interploidy and interspecies hybridization barriers are affected by genome dosage, multiple dosage-sensitive genetic mechanisms, inclusive of imprinting, have been proposed [16,45,51,52,56,58]. The differential dosage hypothesis [51] attempts to unify these various proposals by postulating that any differential parental contribution of a dosage-dependent regulator of viability can affect the triploid block. Such differential contributions could derive from gene expression in the maternal sporophyte, gametophytes (embryo sac or pollen), or endosperm, or from parentally skewed expression in embryo tissue. There is, as of yet, no practical verification of these possibilities as contributors to postzygotic isolation. Nevertheless, genes exhibit parent-dependent effects on seed development by mechanisms other than imprinting. For instance, altering the genotype of the female gametophyte can result in growth and differentiation of the seed coat in the absence of fertilization [59–65]. Genes expressed in the seed coat, such as the *TRANSPARENT TESTA GLABRA2* (*TTG2*) transcription factor, control seed development and coordinate the growth of the offspring and the integuments, a maternal organ in the *Arabidopsis* seed [66]. Ultimately, the molecular identification of genes that affect the triploid block will ascertain whether multiple seed compartments and generations can affect interploidy seed lethality.

## Results

### Arabidopsis Ecotypes Vary in Paternal Excess Tolerance

**Figure 1:**
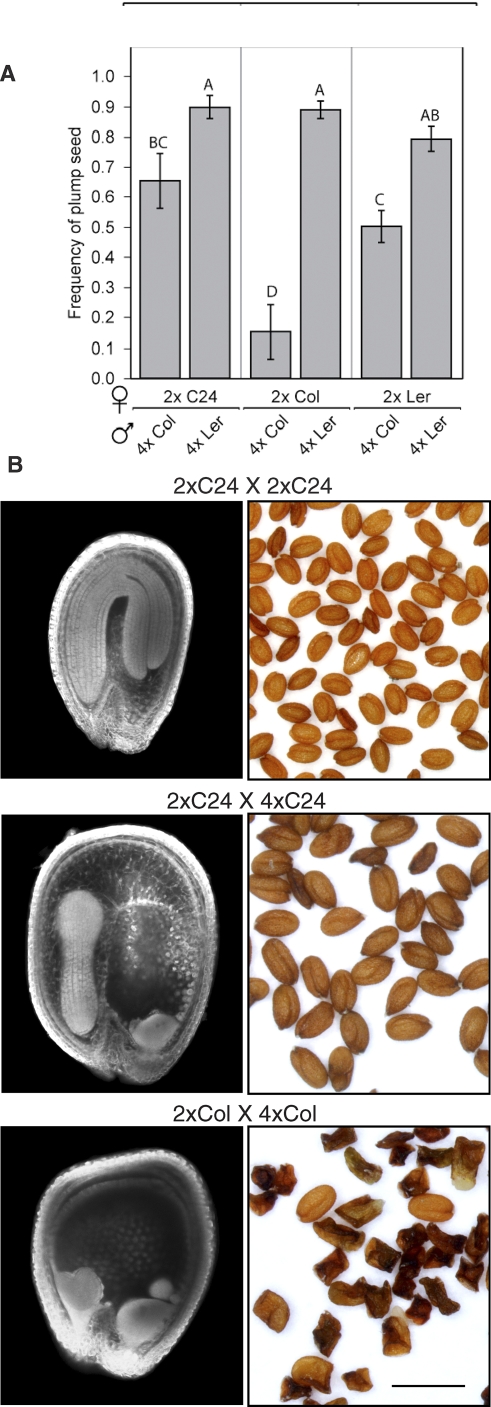
Arabidopsis Strains Vary in the Frequencies of Seed Death in 2x × 4x Crosses **(**A) Genetic background determines seed survival in interploidy crosses. Diploids from *Arabidopsis* accessions Ler, Col-0, and C24 were crossed as seed parents to tetraploid derivatives of Ler and Col. The frequency of plump seeds in the progeny of these crosses (*y*-axis) was determined and the mean for each cross is given along with the standard error. Shared letters above the histogram indicate genotypes that were not different by Student *t*-test at *p* < 0.05. (B) Confocal laser-scanning photomicrographs of developing seeds at 7 DAP (left) and photomicrographs of mature seeds (right) from 2x C24 × 2x C24, 2x C24 × 4x C24, and 2x C24 × 4x Col. Adding paternal genomes by crossing with a 4x pollen parent caused a delay in embryo development and increased the size of endosperm and seeds. In the crosses to 4x Col, overgrowth of the endosperm and aberrant cellularization are evident in the embryo-surrounding region and peripheral endosperm (left panel), and most seeds abort (right panel).

To study the mechanism of interploidy seed failure, we investigated the effect of crosses generating paternal excess in different ecotypes of the model plant A. thaliana. Crosses between diploid (2x) seed parents and tetraploid (4x) Ler pollen parents within the Ler or C24 ecotypes produced mainly plump seed (Figure 1 and [42]). In contrast, crosses between 2x Col, Ler, or C24 seed parents and 4x Col pollen parents produced significantly more shriveled seed (Figure 1A). In a previous study, microscopic observation of developing seeds in crosses between 2x and 4x C24 plants showed that the paternal excess phenotype is characterized by overgrowth of endosperm accompanied by delay of endosperm cellularization [42]. In the same study, crosses between 2x C24 seed parents and 6x Col pollen parents produced more extreme phenotypes, including a failure of cellularization, arrest of embryos at the globular to heart stage, and seed abortion. We compared developing seeds from 2x × 4x crosses in the Col genetic background to seeds from 2x × 2x and 2x × 4x crosses in C24. Mature seeds from these three crosses along with confocal images of developing seeds are shown in Figure 1B. At 7 days after pollination (DAP), seeds from 2x × 2x crosses contained embryos at the bent cotyledon stage, and the endosperm was completely cellularized. The 2x × 4x seeds in C24 at 7 DAP had embryos at torpedo stage, cellularized peripheral endosperm, and uncellularized chalazal endosperm, showing a delay of embryo development and endosperm cellularization. Seeds from 2x × 4x crosses in Col exhibited an even more severe delay. Col 2x × 4x seeds did not have cellularized peripheral endosperm, and embryos had not passed heart stage even at 8 DAP. Therefore 2x × 4x crosses in Col displayed more features of the previously characterized lethal paternal excess phenotype than 2x × 4x crosses in C24.

The frequency of seed failure was influenced both by the seed parent and the pollen parent. For each of the three ecotypes used as seed parents, crosses were made to Ler and Col tetraploids. Crosses to 4x Col produced more failed seeds, regardless of the maternal genotype (Figure 1A). The highest frequency of F1 lethality (∼80% failure, Figure 1) was observed in 2x × 4x crosses within the Col ecotype. This ruled out divergence-derived genotypic incompatibility, such as hybrid incompatibility [67], as the cause of interploidy seed failure in crosses to 4x Col pollen-parents. Furthermore, the greater survival of seeds derived from 2x Ler relative to 2x Col in crosses to 4x Col pollen parents indicated that a genetic analysis of the maternal control of interploidy incompatibility was possible.

### Identification of QTL Responsible for Overcoming Paternal Excess

**Figure 2:**
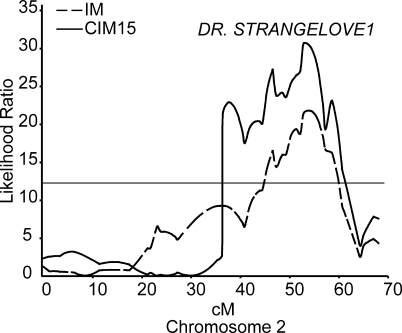
Identification and Localization of the *Dr. Strangelove1* (*DSL1*) QTL Affecting the Paternal Excess Interploidy Hybridization Barrier in Ler × Col-0 RIL Likelihood ratios (LRs; *y*-axis) for the presence of a QTL determined by interval mapping (IM; broken line) and composite interval mapping (CIM; solid line) affecting the frequency of plump seed in crosses of Col × Ler RIL to tetraploid Col. The position on A. thaliana chromosome 2 is presented, in centiMorgans, on the *x*-axis. The estimated *p* < 0.05 threshold at LR = 12.6 (horizontal line) was determined by permutation tests.

To map the loci responsible for the differences between the two ecotypes at higher resolution, recombinant inbred lines (RILs) derived from Ler and Col [68] were crossed to 4x Col pollen parents. The rates of seed failure varied among the RIL and differed substantially from the Ler and Col parents (Figure S1). QTL detection and localization by composite interval mapping [69] identified a major locus affecting seed survival in 2x × 4x crosses on chromosome 2 (Figure 2; permutation adjusted *p* = 0.001). We called this QTL *Doctor Strangelove1* (*DSL1*; [70]). The chromosomal position of *DSL1* was estimated to lie between markers gpa1 and bio2b by bootstrap resampling of the data at a 0.90 confidence interval (a table containing the RIL accessions, marker genotypes, and phenotypes is available in Table S1). Ler alleles at this locus were estimated to improve the chance of seed survival in homozygous RIL by 17%, and accounted for 23% of the phenotypic variance in seed survival observed in this population.

The large effect of *DSL1* was confirmed using crosses with chromosome substitution strains (CSS), in which single Ler chromosomes were introgressed into the Col background [71]. CSS lines were crossed to 4x Col, and the percentage of plump to shriveled progeny were compared to those from 2x × 4x Col crosses (Figure S2). The line CSS2, which is homozygous for Ler chromosome 2, including the *DSL1* locus, displayed a 38% increase in crossing success relative to the 2x × 4x Col controls. No other Ler chromosome substitutions significantly improved crossing success. Chromosome 2 was further investigated using stepped aligned inbred recombinant strains (STAIRS), which are homozygous for a single chromosomal segment of Ler in an otherwise Col background [71]. Seeds from crosses between STAIRS carrying different segments of Ler chromosome 2 and 4x Col pollen parents were assayed for viability using germination tests. Germination frequencies of seeds from the STAIRS used as seed parents and from Col controls were determined (Table S2). Using this method, upper and lower boundaries for the position of *DSL1* were established at nga1126 and nga168, which overlaps with the confidence interval for the QTL determined using the RIL population. Thus, we were able to confirm both the large effect and the position of *DSL1*.

**Figure 3:**
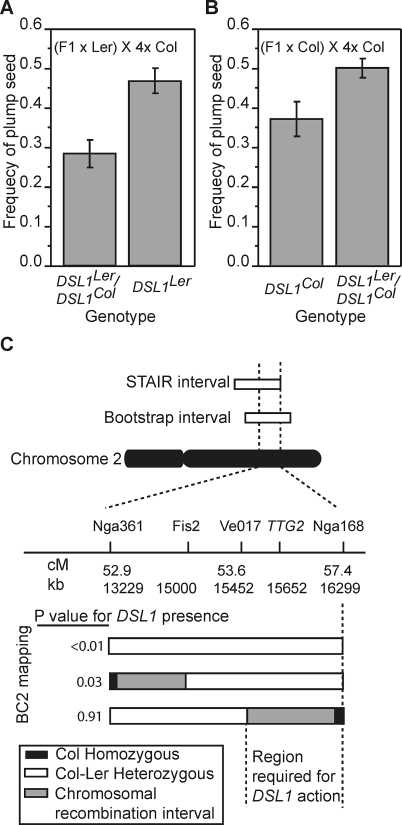
Fine Mapping of *DSL1* (A and B) The mean frequencies of plump seed (*y*-axis, error bars indicate the standard deviations) in BC1 families from F1 × Ler (A) and F1 × Col (B) BC1 families genotyped at the FIS2 marker (*x*-axis) and crossed to 4x Col. Heterozygotes and homozygotes were different as determined by *t*-test at *p* < 0.05 in both populations. (C) Illustrations of the genotype of BC2 families (grey, white, and black boxes) segregating for chromosomes recombinant within the *DSL1* interval. The *p*-values to the right of the illustrations indicate whether segregation of that interval resulted in segregation of the DSL1 QTL as determined by *t*-tests comparing the mean proportion of plump seed in progeny of crosses between 4x Col and BC2 seed-parents heterozygous or homozygous for segregating molecular markers.

To further refine the estimate of the *DSL1* position and investigate the mode of inheritance for *DSL1*, the frequency of shriveled seed in the progeny of backcross (BC) generations was determined in crosses to 4x Col. Two BC1 (F1 × parental line) populations were generated by backcrossing a Col × Ler F1 to both Ler and Col diploids. The frequency of plump seed was compared to the genotypes of markers located in the *DSL1* interval. In both BC1 populations, crossing success increased with increasing dosage of *DSL1^Ler^* alleles (Figure 3A and 3B). The genotype at *DSL1* accounted for 16% and 30% of the phenotypic variation in seed set observed in the F1 × Col and F1 × Ler BC populations, respectively. The change from a heterozygous genotype at *DSL1* to homozygous *DSL1^Ler^* increased surviving interploidy seeds by 19.2% in the Ler BC1, whereas the change from heterozygous *DSL1* to homozygous *DSL1^Col^* decreased seed survival by 15.0% in the Col BC1 population. These estimates of the magnitude of *DSL1*'s effect on seed survival are similar to those made in both the RIL and chromosome substitution populations, confirming both the position and large effect of *DSL1*. In addition, the comparisons of the heterozygote to either homozygote demonstrate that the allelic dosage at the *DSL1* QTL additively affects viability.

Individual BC1 (F1 × Col) plants with recombination breakpoints within the *DSL1* interval were backcrossed to 2x Col to generate BC2 families. BC2 individuals were genotyped at four markers within the *DSL1* QTL: nga361, *FIS2*, Ve017, and nga168. Progeny from BC2 individuals crossed to 4x Col pollen parents were phenotyped. Statistical significance of *t*-test comparisons between Col homozygotes and heterozygotes at the *FIS2* marker are presented for each BC2 family in Figure 3C. Inheritance of the Ler or Col alleles of a chromosomal region spanning markers nga361 through Ve017, including the *FIS2* locus, was not sufficient to change interploidy crossing success. Longer introgressed segments, and segments segregating for the region containing *FIS2* and extending through nga168, however, were sufficient to produce the *DSL1* effect. Taken together, the RIL, STAIR, and BC2 mapping data suggest that the *DSL1* QTL resides between markers Ve017 and nga168.

None of these genetic analyses could distinguish between QTL caused by gene expression in the female sporophyte and postmeiotic gene expression in the female gametophyte or fertilization products. This was addressed using the progeny of a F1 (Ler × Col) × 4x Col cross. Markers linked to *DSL1* should depart from the expected ratio 1:1 of Ler:Col in the progeny of this cross if *DSL1* acts via expression in the female gametophyte or fertilization products. By contrast, no distortion of allele frequency is expected if *DSL1* acts via sporophytic expression, such as via the seed-parent's vegetative organs, flowers, or in the seed coat. Progeny from F1 × 4x Col (*n* = 164) were typed for the same markers used in Figure 3C that span the *DSL1* interval. Chi-square tests did not detect a significant departure from the 1:1 null hypothesis for any of the markers. The lack of distortion at markers linked to *DSL1* argues against rescue of lethality due to postmeiotic expression. The presence of genetic variation sufficient to rescue seed lethality when contributed maternally, but not associated with transmission ratio distortion at linked markers, indicates that the genotype of the maternal sporophyte is critical for the interploidy hybridization barrier.

### *DSL1* Participates in an Epistatic Network

Genome-wide scans for epistatic interactions with *DSL1* were carried out by regression analysis. Significance thresholds for the detection of epistasis were set by permutation and estimation of the effect of all marker pairs plus their interaction in each permuted set. Regressions using two markers and their interaction with combined effects that exceeded this threshold were identified (for a complete table, see Table S3). These putative epistatic interactions were reexamined by multiple regression to ensure that the interaction component significantly improved the fit of the model to the data.

**Figure 4:**
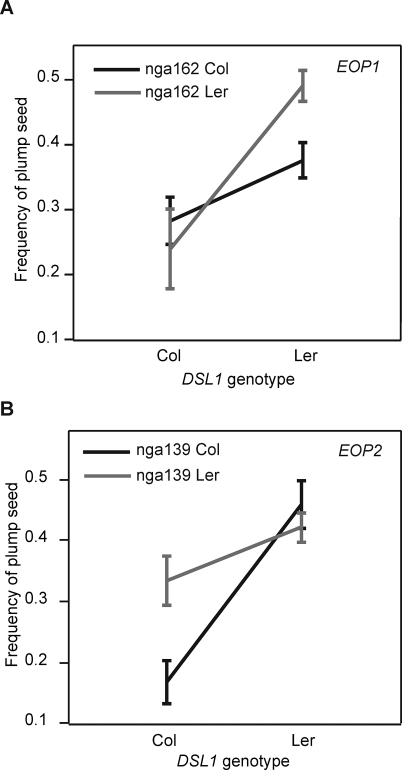
*DSL1* Interacts with at Least Two Loci to Control the Interploidy Hybridization Barrier The genotype at marker g17288, which had the highest likelihood ratio in the RIL population, was used for the *DSL1* genotype on the *x*-axis. The *y*-axis corresponds to the mean frequency of plump seed for each genotype. Error bars indicate the standard deviation from the mean. The grey and black lines correspond to the Ler and Col alleles of nga162 at the position of *EOP1* on chromosome 3 (A) and nga139 at the position of *EOP2* on chromosome 5 (B).

Two loci, *EPISTASIS OVERCOMES PATERNAL EXCESS1* (*EOP1*) and *EOP2*, comprising multiple markers on chromosome 3 and chromosome 5, respectively, affected seed survival when considered in conjunction with the genotype at *DSL1* (Figure 4 and Table S3). The effect of the *DSL1*-linked marker g17288 on the percentage of surviving seeds in RIL × 4x Col crosses displayed a significant interaction with the *EOP1* and *EOP2* linked markers nga162 and nga139, respectively. Regression models of the *DSL1-EOP1* and *DSL1-EOP2* interactions were significant at a permutation-test–adjusted *p* < 0.01, and the sum of the interaction terms accounted for as much as 15% of the phenotypic variation in the RIL population. The Ler alleles of either *EOP1* or *EOP2* increased the proportion of plump seed. In *DSL1^Ler^* plants, *EOP1^Ler^* increased the proportion of plump seed in crosses to 4x Col (Figure 4A), whereas in the *DSL1^Col^* background, the *EOP1^Ler^* allele caused a slight, but insignificant, increase in crossing success. The effect of the other epistatic locus, *EOP2*, on the frequency of seed abortion is only observed in the *DSL1^Col^* background. The combination *EOP2^Ler^* and *DSL1^Col^* resulted in a higher frequency of plump seed formation. Estimated jointly, *EOP1*, *EOP2*, and *DSL1* plus their interactions accounted for over 40% of the phenotypic variance in seed viability in Ler × Col RILs crossed to 4x Col (Table S4).

The effect of *EOP1* on cross fertility was strong in the RIL, so the mode of *EOP1* action was investigated by transmission ratio test. If *EOP1* acts via the genotype of the fertilization products or embryo sac, it should exhibit a distorted ratio in interploidy crosses between Col × Ler F1 hybrids and 4x Col. If, like *DSL1*, the interaction occurs between alleles in the maternal sporophyte, no distortion should be observed. Markers linked to *EOP1*, nga172, and nga162 were genotyped in the F1 × 4x Col progeny. Consistent with a postsegregation requirement for *EOP1* in interploidy seeds, the genotype at nga172 was distorted in triploid (X^2^ *p*-value = 0.0008, observed heterozygotes [Het] = 109, Col = 65 vs. expected Het = 87, Col = 87), but not in diploid progeny (*p*-value > 0.05; Het = 47, Col = 54). A similar trend was observed at nga162, but it was not significant. The detection of interploidy-dependent transmission ratio distortion confirms the presence of a QTL on chromosome 3 that affects interploidy viability via expression in the female gametophyte, endosperm or embryo.

### *TTG2* Regulates the Interploidy Barrier

We sought to determine which gene within the *DSL1* interval could be responsible for the phenotypes observed. The imprinted gene *FERTILIZATION INDEPENDENT SEED2* (*FIS2*) is near the *DSL1* interval, and the Col allele has a 180-bp in-frame deletion in the coding region with respect to Ler and C24 [65]. Another candidate gene in the *DSL1* interval is *TTG2*. *TTG2* encodes a WRKY transcription factor that controls epidermal cell fate with pleiotropic effects on seed development and trichome production [72]. Seeds from mothers homozygous for *ttg2* loss-of-function mutations have a pale seed coat because the innermost layer of the seed coat (the endothelium) fails to accumulate pigments; in addition, the epidermal layer of the outer integument does not produce mucilage [72]. *ttg2–1* mutants produce small seeds exhibiting reduced cell elongation in integuments, and precocious endosperm cellularization [66]. The mutants also have reduced leaf trichome density and decreased trichome branch number [72,73].

As the *DSL1* QTL was additive and sporophytic (Figure 3), we tested *FIS2* and *TTG2* for maternal sporophytic effects on interploidy lethality. Homozygous loss-of-function mutants at *FIS2* are difficult to obtain because *fis2* ovules abort. If *FIS2* encodes *DSL1*, a heterozygote carrying a *fis2* mutant allele should change the viability of all ovules present on heterozygous sporophytes (plants) irrespective of whether they inherited the mutant or wild-type allele. No effect on the survival of wild-type ovules in crosses to 4x Col was observed for heterozygotes of an insertional allele*, fis2–8*, introgressed into the Col background (Table S5). This result is consistent with our observation that segregation of the Ler and Col alleles at *FIS2* was not sufficient for *DSL1* detection in a BC2 family (Figure 3C) and that the mode of inheritance for *DSL1* is maternal sporophytic rather than the imprinted/gametophytic inheritance of the *fis2* seed phenotype [64,65,74].

**Figure 5:**
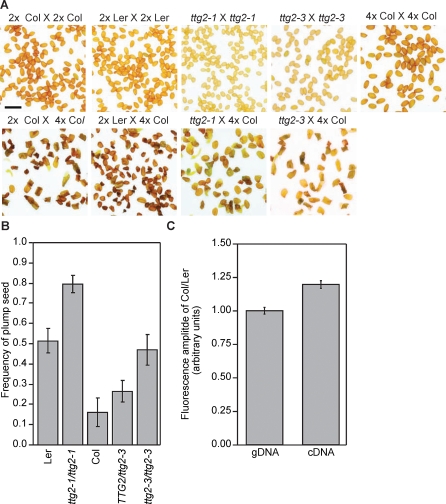
*TRANSPARENT TESTA GLABRA2* Affects Interploidy Hybrid Viability (A) Photomicrographs of the mature seed phenotypes of selfed progeny and crosses with 4x Col for wild types and *ttg2* mutants in the Ler and Col backgrounds. (B) The frequency of plump seed (*y*-axis) following crosses between each of the indicated maternal genotypes (*x*-axis) and tetraploid Col pollen parents. Error bars indicate the standard error of the mean (s.e.m.). (C) The Col allele of TTG2 is more highly expressed than the Ler allele in F1 hybrids. The ratio of fluorescence from the incorporation of a labeled G or C residue at a SNP differentiating the Ler and Col allele of TTG2 was determined in the amplified products from genomic DNA (gDNA) and cDNA isolated from Ler × Col F1 hybrids. The *y*-axis values are scaled to the ratio determined for genomic DNA, and the standard errors are displayed. Student *t*-tests indicate that the Col allele contributed a greater proportion to the cDNA than was present in the 1:1 mix in genomic DNA.

A trichome density QTL in the Ler × Col RIL population was detected in previous studies, consistent with a weak loss-of-function allele of *TTG2* in Ler [75–77]. The effect of *TTG2* on seed rescue was tested by crossing loss-of-function *ttg2* mutants isolated in both Ler and Col backgrounds to 4x Col pollen parents. The *ttg2–1* mutant was previously isolated in the Ler background [72]. We also identified a novel allele in the Col background, *ttg2–3* (SALK_148838), created by a T-DNA insertion in the 5′ untranslated region (UTR) of the *TTG2* gene. Like *ttg2–1*, *ttg2–3* results in few and aberrant trichomes [73] and a loss of seed coat pigmentation (Figure 5A). We found that mature, desiccated *ttg2–3* seeds were 94.7% the length and 96.0% the weight of Col seeds (Table S6). The seeds of *ttg2–1* seeds, by comparison, were 83.5% the length and 80.5% the weight of wild-type Ler*.* Mature seeds produced by each of the four seed parents that either self-pollinated or crossed with 4x Col pollen parents are shown in Figure 5A. Along with plump and shriveled seed, we observed some seeds with aberrant development and maturation. Some embryos had emerged from a ruptured seed coat but, when sown on filter paper, were able to continue growth and form seedlings (unpublished data). These aberrant seeds were classed as plump seeds when estimating viability as they contained viable embryos of substantial size. Frequencies of plump seed are shown in Figure 5B, and data and *t*-tests in Table S7. When *ttg2–1* mutants, in the Ler background, were crossed to 4x Col pollen parents, over 78% of seeds were plump (*n* = 136). This was significantly different from wild-type Ler seed parents, which produced 52% (*n* = 289) plump seed in crosses to 4x Col. Similarly, the percentage of plump seed increased from approximately 14% (*n* = 198) in crosses between 2x and 4x Col to 64% (*n* = 111) when *ttg2–3* mutants were substituted as seed parents. *TTG2*/*ttg2–3* heterozygotes exhibited a slight increase in fertility, but the trend was not significant (Figure 5B, *t*-test *p* > 0.05). Thus, loss-of-function alleles at *TTG2* reduced the interploidy hybridization barrier. Consistent with *TTG2* encoding the *DSL1* QTL and the Ler genome encoding a weak allele of *TTG2*, the degree of rescue provided by a loss-of-function allele in Col was many-fold greater than in Ler.

No polymorphisms between the Ler and Col coding sequence of TTG2 were found. We therefore sought to determine whether an expression level polymorphism differentiates the alleles. Single nucleotide polymorphisms (SNPs) present in the TTG2 transcript were identified in the data from chip-based resequencing of A. thaliana [78]. A SNP present in the 5′ UTR of the full gene (PERL030427) was confirmed by sequencing of PCR reactions using genomic DNA from the Ler and Col ecotypes as template. The proportion of each allele was measured in the amplified products of DNA and cDNA prepared from Ler × Col F1 hybrids. By measuring the proportion of the two alleles that accumulated in an F1 hybrid, the two alleles were tested in an identical regulatory environment at equal gene dosage, and any differential allelic contribution must result from a *cis*-regulatory difference between the two alleles. The proportion of TTG2 mRNA contributed by the Ler allele to the reverse transcription PCR products was approximately 20% less than genomic DNA samples (*t*-test *p*-value < 0.001) (Figure 5C). This indicated that the Ler allele accumulates less *TTG2* message, consistent with the Ler encoding a weak *TTG2* allele and the relative effects of the *ttg2–1* and *ttg2–3* knockout alleles in improving interploidy cross fertility.

The hybridization-based resequencing data did not clearly indicate any polymorphisms that might explain the difference in expression. To determine what, if any, putative *cis*-regulatory polymorphisms exist, the DNA sequence immediately upstream of the presumptive transcriptional start site was determined in Ler. At the −53 position, relative to the start of the longest *TTG2* cDNA, and 11 bp downstream of the previously identified *Werewolf*, *Glabrous1*, and *Transparent Testa2* transcription factor binding sites [73], Ler contains a 7-bp insertion of the sequence AGACCAA between the nucleotides ACGTTCAACGAGTGTCCAT and a 26-bp stretch of pyrimidines, beginning with CTCCCTC. This insertion is 43 bp 5′ of the presumptive TATA box of the *TTG2* promoter. Consistent with the presence of an insertion, the hybridization-based resequencing data [78] show hybridization failure in microarrays hybridized with Ler, but not Col, labeled genomic DNA beginning with probes 8 bp upstream of the insertion site (http://signal.salk.edu/perlegen.html). Amplification of genomic DNA from Col and Ler using PCR primers flanking the insertion confirmed a fragment-length polymorphism distinguishing Col and Ler that could be resolved on an agarose gel (unpublished data).

### Cellularization of the Peripheral Endosperm Is Associated with Rescue of Interploidy Seeds

**Figure 6:**
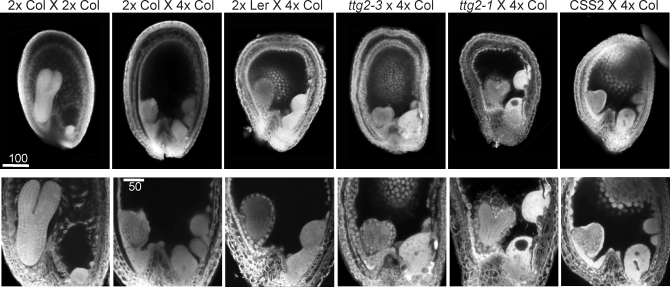
Confocal Photomicrographs of the Endosperm Cellularization Phenotypes of Developing Seeds from 2x × 2x and 2x × 4x Crosses at 6 DAP The first row shows full seeds, and the second row shows close-ups of the embryo and surrounding region and the chalazal endosperm. All seeds are oriented with the embryo on the left and chalazal endosperm to the right.

**Figure 7:**
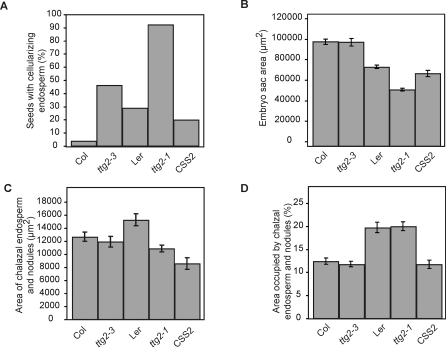
Endosperm Cellularization Is Associated with Interploidy Cross Success (A) Diploid seed parents were crossed to tetraploid Col. The percentage of seeds with cellularizing endosperm at 7–8 DAP (*y*-axis) was quantified in cleared seeds. The genotypes of the diploid seed parents are indicated on the *x*-axis. (B) Crosses and seed clearing performed as in (A), but the plan area of the embryo sac was recorded. Means and standard deviations are displayed. (C) Crosses and seed clearing performed as in (A), but the plan area of the chalazal endosperm and endosperm nodules were recorded. Means and standard deviations per seed are displayed. (D) Data from (B) and (C) were used to calculate the percentage of the embryo sac area occupied by the chalazal endosperm and nodules in each seed. Means and standard deviations per seed are displayed.

We next examined developing seeds from crosses between Col, Ler, *ttg2–3*, *ttg2–1*, or CSS2 seed parents and 4x Col pollen parents. Phenotypes associated with lethality and rescue were observed by confocal and differential interference microscopy. Confocal images of seeds at 6 DAP are shown in Figure 6. All crosses displayed features we have previously observed in seeds with paternal excess, including enlarged chalazal endosperm and nodules, and delays of embryogenesis and of endosperm cellularization. We examined the size of the chalazal endosperm and nodules, since these were previously observed to be larger in seeds with lethal than with viable paternal excess [42]. Maximum cross-sectional areas of the embryo sac and of chalazal endosperm plus nodules were measured on digital photographs of cleared whole-mount seeds. We found no clear relationship between embryo sac size at 7 DAP (the latest time point when cleared seeds could be imaged) and viability, as *ttg2–1* × 4x Col seeds had smaller embryo sacs than 2x Ler × 4x Col, but *ttg2–3* × 4x Col embryo sacs were a similar size to those of 2x Col × 4x Col (Figure 7B). This ruled out reduction of integument growth as a general factor in seed rescue. Neither was a restriction in size of the chalazal endosperm and nodules required for seed fertility, as Ler × 4x Col seeds had larger chalazal endosperm and nodules than Col × 4x Col seeds, in both absolute and relative terms, at 7 DAP (Figure 7C and 7D). In fact in *ttg2–1 ×* 4x Col, these structures occupied nearly twice the relative area as in Col × 4x Col. The relative area of the seed cavity occupied by chalazal endosperm and nodules was greater in Ler and *ttg2–1* than in Col, *ttg2–3*, and CSS2, demonstrating additional variation in endosperm developmental programs in Ler and Col.

The frequency and timing of endosperm cellularization was determined by examining confocal micrographs of developing seeds. The frequency of endosperm cellularization was consistently increased in all comparisons that improved interploidy crossing success (Figure 7A). By 8 DAP, nearly all *ttg2*-1 × 4x Col seeds contained cellularizing endosperm, whereas only some Ler × 4x Col showed signs of endosperm cellularization. Similarly, seeds from *ttg2–3 ×* 4x Col were intermediate in the frequency of cellularization, but hardly any 2x Col × 4x Col seeds showed signs of cellularization. CSS2 × 4x Col likewise was more frequently cellularized at 8 DAP than 2xCol × 4x Col. Similar results were obtained from observations at 7 DAP and 6 DAP (unpublished data). We concluded that *TTG2* action in the sporophyte delays endosperm cellularization in paternal excess crosses, and this hinders embryo development.

## Discussion

Postzygotic hybridization barriers restrict gene flow and isolate populations from one another. It has long been known that shifts in ploidy can affect postzygotic lethality and create isolated populations of different ploidy. This kind of F1 lethality can occur in the absence of any allelic diversity. The presence of such barriers has been proposed as one of the mechanisms by which shifts in ploidy can potentiate the process of speciation [9,21,79]. Yet, considerable variability in this trait exists within species and between species [9,41]. We investigated the molecular mechanism responsible for variation in the lethality of interploidy crosses in *Arabidopsis*.

The genotype of both the paternal and maternal lineages caused variation in the postzygotic lethality of 2x × 4x crosses (Figure 1). In agreement with previous studies [42,55], interploidy crosses using accessions Ler and C24 produced a majority of live seed whether within or between genotypes. Crosses using diploid Col seed parents and either Ler or C24 tetraploid pollen parents also resulted in a majority of well-formed plump seeds. When Col tetraploids were used as pollen donors, however, a dramatic increase in the frequency of failed seeds occurred in crosses to all three maternal backgrounds. Importantly, the loss of viability was not due to genetic incompatibilities between accessions, as the least compatible cross was 2x Col × 4x Col in which identical genotypes were contributed by both parents at different dosages. The expression of variation via either parent indicates that the genetic basis must either be dosage sensitive, i.e., additive, or result from independent maternal and paternal effects. Like earlier studies [42,55], the initial crossing design could not distinguish between additivity, parental effects mediated by parent-dependent genomic imprinting, or maternal sporophytic, cytoplasmic, or gametophytic effects. Any or all of these could contribute to interploidy lethality [51].

### *Dr. Strangelove1* and an Epistatic Network Control Interploidy Lethality

A single QTL, called *Dr. Strangelove1*, was detected using the fertility of Ler × Col RIL crossed to 4x Col as a quantitative trait (Figure 2). The effect of the genotype at *DSL1* was also observed when BCs of the F1 to both Ler and Col were crossed to 4x Col, indicating that the phenotype was sensitive to the dosage of *DSL1* alleles in the maternal parent. The maternal contribution of Ler alleles was sufficient to rescue part of the lethality, and alleles were not subject to transmission ratio distortion in F1 × 4x Col crosses. Thus, the genotype of the maternal sporophyte was likely responsible for the action of the QTL. This validates the proposition that gene regulation during all life cycle stages must be considered as possible explanations for variation in seed fitness, parent-of-origin effects, and hybridization barriers, and not only genomic imprinting in the zygotes [51]. Indeed, a requirement for the coordination of the maternal sporophyte and endosperm development suggests that parent–offspring conflict could cause the observed variation.

Two additional QTL, *EOP1* and *EOP2* (Figure 4), interact with the genotype of markers linked to *DSL1*. This limited genetic network modulated seed lethality and contributed approximately 20% of the phenotypic variance in seed survival in the RIL population. A similar, but more extensive, network of epistatic loci has been found to affect variation in A. thaliana accessions F1 hybrid fitness in crosses to A. arenosa (C. Josefsson, B. Dilkes, and L. Comai, unpublished data). Such “intragenomic” epistasis, distinct from epistasis expressed due to the divergence between species, may be a general feature of hybridization barriers that arises due to the dosage-sensitivity of the genes responsible for F1 lethality. Dosage-sensitive genes are more likely to encode members of signaling cascades and protein interaction networks [80]. Such networked gene products would be more susceptible to interlocus variation and epistasis. Moreover, selection on a trait affected by a network could result in the accumulation of mutations at multiple interacting genes.

We made no estimates of the confidence intervals for the positions of *EOP1* or *EOP2*. Nevertheless, the finding that *TTG2* (discussed below) could affect the interploidy barrier suggests that loci with demonstrated genetic interactions with *TTG2* such as the leucine-rich receptor-like kinase *Haiku2* (*IKU2*) [66] may also possess the ability to regulate the ploidy barrier. Similarly, upstream regulators of *TTG2*, such as the transcription factors *TTG1* and *TT2*, which are required for *TTG2* expression [81], might also modulate interploidy lethality. Interestingly, *IKU2* and *TTG1* map to the same chromosome arms as *EOP1* and *EOP2*, respectively.

### TTG2 and the Control of Interploidy Lethality

The transcription factor *TTG2*, which controls seed development via expression in the maternal sporophyte, was located within the chromosomal interval containing *DSL1* [66,72]. Seeds from *ttg2–1* mutant sporophytes possess defects in both sporophytic (seed coat differentiation) and zygotic (precocious endosperm cellularization) traits. We propose that allelic variation at *TTG2* could explain the *DSL1* QTL. Loss-of-function mutations in *TTG2* isolated from both the Ler and Col backgrounds improved interploidy crossing success (Figure 5B). *DSL1* is an additive QTL, yet *ttg2–1* has been shown to be recessive for most seed size traits [66] and is reportedly recessive for trichome density [72]. Nevertheless, at least one comparison between *ttg2–1*/+ and +/+ maternal crosses, seed width, was reported as significantly different (see Table 1 in [66]), demonstrating that a single wild-type *TTG2* locus can be insufficient to support completely normal seed growth in balanced crosses as well.

Additional phenotypes are consistent with Ler encoding a weak *TTG2* allele. A QTL affecting reduced trichome density and linked to *TTG2* has been described previously [75–77]. The effect of this QTL on trichome density is additive [77], just like *DSL1* (Figure 3). Seed color in Ler is also lighter than Col, as would be predicted [82]. These data are all consistent with the Ler allele at *DSL1* encoding a hypomorphic allele of *TTG2*. The alleles have not been transgenically tested for differential effects on the hybridization barrier, but a *cis*-regulatory difference in expression of the alleles was detected in Ler/Col heterozygotes. The difference in transcript level for this transcription factor is consistent with the type of variation that can underlie QTL function. Regardless, the large single-gene effect in *ttg2–1* and *ttg2–3* mutants observed here demonstrates that reproductive isolation by ploidy and fertility restoration can be mediated by single mutations expressed in the sporophyte.

### Control of Cellular Differentiation in the Endosperm and the Mechanism of DSL1- and TTG2-Mediated Fertility

The earliest evidence of aberrant development in the progeny of paternal excess crosses was visible in the endosperm [42]. Endosperm failed to progress from the free nuclear proliferation typical of early development to cellularized mitotic proliferation, which normally occurs at the globular to heart transition of embryogenesis. The lack of endosperm cellularization in 2x Col × 4x Col crosses is similar to that observed in A. thaliana × A. arenosa crosses [49] and the heterochronic shift described in fertilized seeds of mutants affected in the *Arabidopsis* orthologs of Polycomb-Repressive Complex 2 subunits [59–65,83]. Paternal excess crosses of diploid Ler or Col and tetraploid Col differed in their respective endosperm growth phenotypes. Cellularized peripheral endosperm was observed more frequently in crosses to Ler seed parents than in Col seed parents (Figure 7A). Rescue of interploidy lethality by substituting the chromosome containing *DSL1* from Ler into a Col background was also associated with more frequent observation of cellularized peripheral endosperm in developing seeds.

*TTG2* was identified as an effector of plant development. The *ttg2–1* allele in the Ler background has a maternal effect on seed size and induces early cellularization of the endosperm in balanced crosses [66,84]. Loss of *TTG2* from diploid maternal sporophytes can result in seed failure due to an interaction with the *haiku2–1* (*iku2–1*) mutation [66], which also decreased seed size and induced early cellularization of the endosperm. This demonstrated that proper *TTG2* function can be required for viability. Similar to the effects in balanced crosses, *ttg2–1* × 4x Col crosses also produced seeds with precocious endosperm cellularization as compared to wild-type Ler × 4x Col (Figure 7). This rescue of interploidy fertilization by maternal transmission of *TTG2* loss of function alleles, demonstrated here, further supports the placement of this gene in a pathway coordinating the growth of the maternal tissues and fertilization products.

Rescue of lethality by *DSL1^Ler^* and *ttg2* mutants was not associated with the resolution of all aberrant features of the endosperm. For instance, neither the size of the chalazal endosperm nor the presence of nodules in the peripheral endosperm was consistently affected by genotypes providing rescue of lethality. Peripheral endosperm cellularization, however, was always greater in backgrounds that increased seed survival. Rather than a suppression of early proliferation of chalazal endosperm hypertrophy, cellularization of the endosperm is likely to be the critical feature in paternal excess interploidy crosses that determines the development of the embryo.

One proposed mechanism for the effect of TTG2 on endosperm cellularization in balanced crosses is that the reduced growth of maternal tissue in *ttg2* mutants restricts embryo sac expansion and promotes endosperm cellularization [85]. Mature seeds produced by a self-pollinated *ttg2–1* mutant are notably smaller than wild type, and *ttg2–3* seeds are slightly smaller. Yet, embryo sacs at the time of endosperm cellularization from *ttg2–3* × 4x Col crosses were no smaller than those of the uncellularized proliferating Col × 4x Col. Thus, loss of TTG2 function in Col appears to promote endosperm cellularization independent of seed or ovule growth. Alternative explanations include a change in signaling molecule release or perception due to loss of TTG2. Proanthocyanidins, which were markedly reduced in *ttg2–1* seeds [66,72], decrease the permeability of the endothelium, the maternal cell layer adjoining the endosperm [82]. Their absence in the endothelium of *ttg2* mutants may allow diffusion of a molecule that promotes endosperm differentiation. Alternatively, *ttg2* mutations may prevent normal differentiation of the endothelium [82], and thereby disrupt the production of a signal from the seed coat to the endosperm that delays cellularization in wild-type plants.

### Evolutionary Consequences of Variation in Interploidy Barriers

A stronger postzygotic hybridization barrier, such as provided by the Col genome, would restrict allele sharing between diploids and tetraploids. In the absence of gene flow, independent adaptation of the two populations and/or speciation would be possible. Even complete interploidy block might not result in polyploid speciation as new alleles arising in diploids can be shared with tetraploid populations via unreduced gametes or recurrent polyploid formation. The relative absence of a crossing barrier, however, would result in more frequent triploid production. Triploids of *Arabidopsis* produce aneuploid swarms that resolve into diploid and tetraploid cytotypes [15,44]. More frequent swarm production would increase the opportunities for karyotype evolution, selection of alleles in novel and unbalanced karyotypes, and both diploid and tetraploid descendants from a triploid would facilitate gene flow between diploid and tetraploid cytotypes. This is expected to strongly inhibit the establishment of new polyploid species, but to increase the chance of finding polyploid cytotypes within a diploid population [1,2,5,9]. Thus far, surveys of the nuclear DNA content from a few hundred *Arabidopsis* accessions have identified only two tetraploids [44,86]. A study utilizing crosses between 288 accessions of *Arabidopsis* identified one additional tetraploid accession on the basis of increased seed lethality in the intercross progeny [67]. Rates of 2n gamete formation in pollen meioses of A. thaliana have been estimated to be an order of magnitude lower than these observed frequencies of tetraploid accessions, at approximately 1/3,000 [87]. Given these low frequencies and the low rate of outcrossing in A. thaliana [88,89], selection on mutations that prevent triploid offspring from unreduced gametes or sympatric tetraploids seems unlikely.

Within-species variation for the ploidy hybridization barrier is not unprecedented (e.g., [41]) and may result from selection on seed performance. The genes that have been hypothesized by some to underlie the interploidy barrier might be under continued selection due to the ability of variation in expression to affect seed fitness [51] or purifying selection to maintain optimal expression regulation [90,91]. Despite these suggestions, it is possible that neutral variation, i.e., of no consequence in balanced crosses, limits fitness in the out-of-balance fertilization products produced by interploidy crosses. Further study of the genes and alleles identified here will be necessary to determine whether they are subject to selection in diploid populations. The identification of *TTG2*, which also determines trichome density, as a determinant of variation in interploidy postzygotic lethality suggests that pleiotropy may confound the interpretation of such results. For instance, in the absence of herbivore pressure, trichome density is negatively associated with *Arabidopsis* fitness, whereas it is beneficial in the presence of herbivores [92]. *TTG2* alleles selected for their effect on trichome density, but neutral with respect to seed fertility in balanced crosses, may result in differences in seed lethality following interploidy hybridization.

### Epigenetics and Development Converge in Plant Hybridization

Previously, it has been shown that disruption of gene silencing via loss of CG methylation can disrupt both interploidy survival [55] and interspecies hybrid survival [49]. The disruption of imprinting in the species hybrids may destroy the coordination of the two maternal programs (those expressed in the seed and those in the integuments), resulting in seed failure. In this sense, intergenerational control of seed development is responsible for maternal control of both the species barrier and ploidy barrier (this study; [48,49]). Loss of imprinting in interspecies hybrids was associated with epigenetic disturbance affecting both heterochromatin and genes [48], and chromatin and DNA methylation disturbances have been noted in interploidy crosses of both *Arabidopsis* and maize [93,94]. In this study, we demonstrate that a compensatory change in the maternal genotype can overcome these epigenetic disturbances.

Although it would appear that there are two, as yet, nonoverlapping mechanisms affecting the strength of the interploidy hybridization barrier, they may have more in common. It is reasonable to propose that the mechanism identified here might interact with targets of CG-dinucleotide methylation that coordinate the growth and development of seeds. For example, it is possible that runaway proliferation in the endosperm caused by misregulation of *MEDEA*, an imprinted gene and known target of CG methylation, could be opposed by reduced *TTG2* function.

## Materials and Methods

### Plant materials and growth.

Tetraploids of *Arabidopsis* ecotypes Col-0 and Ler were produced by submerging seedlings for 2 h in an aqueous solution of 0.1% colchicine (Sigma). Seedlings were subsequently washed in copious quantities of water, and transplanted to soil. Treated plants were grown to maturity and the basal nuclear ploidy of the progeny determined by flow cytometric analyses of nuclear DNA content following isolation of nuclei from leaves or inflorescence buds as previously described [44].

RILs derived from a cross between Col and Ler [68] were provided by the *Arabidopsis* Biological Resource Center (ABRC). STAIRS were provided by Michael Kearsey (University of Birmingham, United Kingdom). A male-sterile C24 A9 barnase line described by Paul et al. [95] was crossed to Col-0 for ten successive generations to produce “Col A9” (gift of Rinke Vinkenoog). Col-0/Ler F1 hybrid individuals were crossed to Col A9 male-sterile plants or to hand-emasculated Ler plants to generate reciprocal BC1 families. Col A9 male-sterile plants were crossed to Ler to produce male-sterile F1 plants used to test transmission ratio distortion in crosses to 4x Col. *ttg2–1* was a gift from David Smyth [72]. The novel allele *ttg2–3* was derived from the SALK insertion line 149938 [96]. The *fis2–8* allele [97,98] was provided by Ramin Yadegari and Kristen Newcomb (University of Arizona, Tucson, Arizona), who backcrossed the mutation in the Col background for seven consecutive generations.

The Col × Ler RIL population was grown in the University of Washington greenhouse with ambient light supplemented to make a 16-h light to 8-h dark day. Temperatures were set for 18 °C at night to 21 °C during the day, but varied with atmospheric conditions. All other experiments were carried out in controlled chamber conditions under 16:8-h light:dark and 21:18 °C day:night temperatures. Crosses for the RIL, BC1, and TRD experiments were performed by emasculating inflorescences by removing all organs from the outer three whorls with jewelers forceps. Crosses to the STAIRS were done by removing only the stamens and pollinating 2 d after emasculation. STAIRS and control crosses were grown at the University of Bath in a Sanyo controlled environment room with a day length of 16 h, at 23:18 °C day:night temperatures. For comparison of *ttg2* mutants and wild type, seeds were weighed with a Mettler UMT2 microbalance (Mettler-Toledo).

### Genetic analyses.

For the analysis of RILs, marker genotypes were obtained from the European Union (EU) Natural program (see also, http://arabidopsis.info/new_ri_map.html). Only genetic markers with data for 95% or more of the lines were used for mapping. Markers with no recombination between them were removed from the analysis using the MapManagerQTX 2.0 package [99]. Markers and genotypes for the RIL used in the analysis can be found as Table S4. Three individuals from each RIL were crossed to 4x Col, and the numbers of plump and shriveled seeds were counted. The frequency of plump seeds for each individual was defined as the trait, and RIL means were computed from at least three replicates. QTL identification and likelihood estimation were performed using the mean RIL values under models 1 and 6 in the QTL Cartographer software package [100]. Statistical thresholds for QTL identification were set by 1,000 permutations according to the method of Churchill and Doerge [101]. Estimates of QTL position in the RIL were made by 1,000 bootstraps with replacement followed by interval mapping [102]. The effects of genomic loci segregating between STAIRS and controls or among BC1 populations on the frequency of plump seed following crosses to 4x Col-0 were tested by *t*-test and regression. Transmission ratio distortion was evaluated by chi-square comparisons of observed allele frequencies to expected (1:1) frequencies in the progeny of crosses of F1 or BC1 individuals heterozygous for informative markers to a 4x Col-0 pollen parent. All regressions for multivariate analyses and epistasis confirmation were performed using the JMP statistics program (Ver. 5.1, SAS institute).

Epistatic QTL were identified using MapManagerQTX by regression using every possible marker pair. Statistical thresholds for epistasis detection were set by 1,000 permutations of the data followed by regression on every possible marker pair. Epistatic interactions were considered putative if they exceeded a chi-square value of 28.6, which corresponded to the permutation-adjusted *p*-value of 0.05. Epistatic interactions were considered confirmed if the interaction term was significant in linear regressions of both markers and the interaction term on the mean RIL data (*y* = M1 + M2 + M1 × M2; where *y* corresponds to the mean plump seed frequency and M1 and M2 are marker genotypes).

Marker genotypes of BC1 and BC2 individuals were determined at nga361, nga168, and nga162 by autoradiography of dried gels following electrophoresis of ^32^P alpha-CTP incorporation-labeled PCR products in 6% polyacrylamide (37:1 acrylamide to bis acrylamide) in 0.5 × Tris borate EDTA by standard protocols [103]. Markers nga172, Ve017, TTG2, and FIS2 were analyzed by photography of ethidium bromide fluorescence from electrophoresis of PCR products in agarose and 0.5 × TBE by standard methods. PCR products from primers amplifying marker Ve017 was digested as recommended by The *Arabidopsis* Information Resource (http://www.arabidopsis.org). All primers are given in Table S8.

### eQTL determination by SNP quantitation at TTG2.

Genomic DNA and total RNA were extracted from diploid Ler, Col-0, and F1 hybrid young leaves. cDNA was generated using the gene-specific primer ttg2_bottomR (CTTTCTCCCTTCGACTCACG) and superscriptII Reverse transcriptase according to the manufacture's instructions (Invitrogen). Primers (ttg25′snpR-ttcaccatcattcacctcca; ttg25′snpF-gatccatccaacgttcccta) spanning the first intron of the TTG2 transcript and flanking the PERL0390427 SNP (http://www.arabidopsis.org;[78]) were used as PCR primers using cDNA and genomic DNA as templates. This produced amplicons of 259 and 443 bp from cDNA and genomic DNA, respectively. Amplified products were run on a 2% agarose gel in 0.5 × TBE and purified using the QIAQUICK gel extraction kit (Qiagen) according to the manufacturer's instructions. DNA sequencing was performed by Davis Sequencing using the ttg25′snpR primer utilized in the amplification. Fluorescence peak amplitudes were recorded for the cytosine and guanine peaks corresponding to the PERL0390427 SNP [104]. The relative contribution of the lesser allele to the total was compared in the genomic DNA and cDNA amplifications by *t*-test. There was no overlap in range between the ratios calculated for amplifications of cDNA and genomic DNA-derived samples.

### Microscopy.

Mature seeds were observed by stereomicroscopy using an Olympus SZ-60 microscope equipped with an annular ring light. Seeds were scored as plump if they contained a visible embryo that occupied more than 20% the normal size, and shriveled if they contained no discernable embryo or an aberrant embryo less than 20% of the size of the seed. Mature seeds were photographed with a Nikon SMZ1500 stereomicroscope using a Nikon Digital Sight DS-U1 camera, and JPG images were processed with Adobe Photoshop. Measurements were taken on digital images of 50 seeds per cross using ImageJ (http://rsb.info.nih.gov/ij/). Developing seeds were cleared in chloral hydrate:water:glycerol (8w:3v:1v), photographed with a Nikon 90i Eclipse microscope using differential contrast optics. and measurements taken on 12–20 seeds per cross using ImageJ. For confocal microscopy, Feulgen-stained seeds were processed as previously described [42] and imaged with an argon ion laser, 488-nm excitation and 515/530-nm emission, using a Nikon C1 confocal microscope system with a 90i Eclipse microscope and EZ-C1 software (Nikon UK). Images were saved as TIFFs and processed with Adobe Photoshop.

## Supporting Information

Figure S1 Distribution of the Percentage of Live Seed for Each Ler × Col RIL (50 MB DOC)

Figure S2 Effect of Chromosomal Substitutions on Plump Seed in STAIR × 4x Col Crosses (1.21 MB AI)

Table S1 Marker genotypes of Col × Ler RIL used in QTL identification (75 KB TXT)

Table S2 STAIRs Localization of *DSL1* Position on Chromosome 2 (107 KB DOC)

Table S3 Summary of Significant Pair-Wise Marker Interactions Affecting Interploidy Lethality in Ler × Col RIL Crossed to Tetraploid Col (105 KB DOC)

Table S4 Main and Interaction Effects for %Live Seed in Col × Ler (37 KB DOC)

Table S5 *fis2–8* Did Not Modulate Viability in Crosses to Tetraploid Col (37 KB DOC)

Table S6 TTG2 Modulates Mature Seed Size in Ler and Col Genetic Backgrounds (29 KB DOC)

Table S7 *TTG2* Modulates Seed Viability in Crosses of Diploid Mutants to Tetraploid Col (37 KB DOC)

Table S8 Oligonucleotide Primers Used in This Study (47 KB DOC)

## Abbreviations

BC: backcross
CSS: chromosome substitution strain
DAP: days after pollination
*DSL1*: *DR. STRANGELOVE 1*
*EOP*: *EPISTATSIS OVERCOMES PATERNAL EXCESS*
*FIST*: *FERTILIZATION INDEPENDENT SEED2*
RIL: recombinant inbred line
SNP: single nucleotide polymorphism
STAIRS: aligned inbred recombinant strain
*TTG2*: *TRANSPARENT TESTA GLABRA2*
