# Supplementary material for: The Maternally Expressed WRKY Transcription Factor TTG2 Controls Lethality in Interploidy Crosses of Arabidopsis
Source: PLoS Biol. 2008 Dec 9;6(12):e308. doi: 10.1371/journal.pbio.0060308 (PMC2596861; doi:10.1371/journal.pbio.0060308)
Supplement: Supplementary file 1 [file pbio.0060308.sg002.pdf]

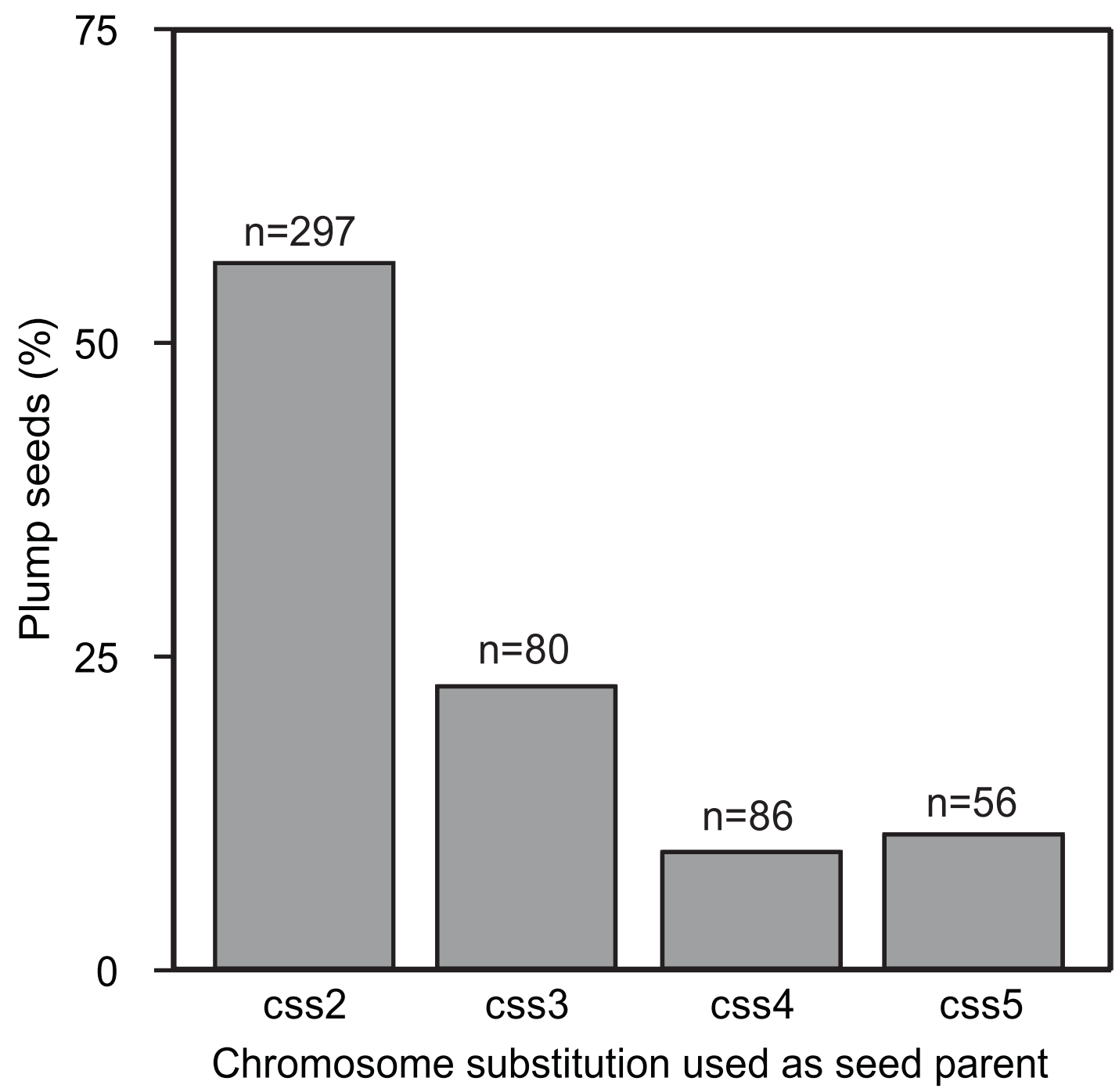

Supplemental figure S2. Substituion of Ler chromosome two in a Col background (Css2) dramatically increases the frequency of live seed in the progeny of crosses to tetraploid Col (Y axis). No other chromosome provided a drarmatic increase over the contol 2x Col X 4x Col crosses (Figure 1).
