# Supplementary material for: The Maternally Expressed WRKY Transcription Factor TTG2 Controls Lethality in Interploidy Crosses of Arabidopsis
Source: PLoS Biol. 2008 Dec 9;6(12):e308. doi: 10.1371/journal.pbio.0060308 (PMC2596861; doi:10.1371/journal.pbio.0060308)
Supplement: Supplementary file 2 [file pbio.0060308.sg001.doc]

Supplemental Figure S1. Distribution of the percentage of live seed for each Ler x Col RIL. The x-axis corresponds to the mean number of plump seed for each RIL. The y-axis corresponds to the number of RIL that fall within the 5% range of each bar on the histogram.

The mean number of plump seed was calculated for each RIL from three replicate individuals crossed to generate a minimum of three siliques each. The distribution of live seed per RIL is summarized in the supplemental figure. A large number of RIL means exceeded the parental range.
