# Supplementary material for: The Maternally Expressed WRKY Transcription Factor TTG2 Controls Lethality in Interploidy Crosses of Arabidopsis
Source: PLoS Biol. 2008 Dec 9;6(12):e308. doi: 10.1371/journal.pbio.0060308 (PMC2596861; doi:10.1371/journal.pbio.0060308)
Supplement: Supplementary file 3 [file pbio.0060308.st002.doc]

**Supplementary table S2.** STAIRs localization of *DSL1* position on chromosome 2

|  |  |  | Marker Genotype^a^ | | | | | | | | | | | | |
| --- | --- | --- | --- | --- | --- | --- | --- | --- | --- | --- | --- | --- | --- | --- | --- |
|  | Marker | | RGA |  | nga1145 |  | MSF3A |  | nga1126 |  | nga361 |  | nga168 |  | T9J23 |
|  | Position (Mb)^b^ | | 255.5 |  | 683.6 |  | 8,110.8 |  | 11,703.6 |  | 13,229.4 |  | 16,298.9 |  | 19,632.9 |
| Line | %germ^c^ |  |  |  |  |  |  |  |  |  |  |  |  |  |  |
| 1240/7 | 32.64 |  | Col |  | Col |  | Ler |  | Ler |  | Ler |  | Ler |  | Ler |
| 1314/9 | 50.22 |  | Col |  | Col |  | Col |  | Ler |  | Ler |  | Ler |  | Ler |
| 1217/4 | 36.52 |  | Col |  | Col |  | Col |  | Ler |  | Ler |  | Ler |  | Ler |
| 1242/12 | 30.61 |  | Col |  | Col |  | Col |  | Ler |  | Ler |  | Ler |  | Ler |
| 1375/5 | 54.92 |  | Col |  | Col |  | Col |  | Col |  | Ler |  | Ler |  | Ler |
| 1274/7 | 69.97 |  | Col |  | Col |  | Col |  | Col |  | Ler |  | Ler |  | Ler |
| 1242/1 | 12.13 |  | Col |  | Col |  | Col |  | Col |  | Ler |  | Ler |  | Ler |
| 1375/12 | 55.99 |  | Col |  | Col |  | Col |  | Col |  | Ler |  | Ler |  | Ler |
|  |  |  |  |  |  |  |  |  |  |  |  |  |  |  |  |
| 1266/3 | 9.89 |  | Ler |  | Col |  | Col |  | Col |  | Col |  | Col |  | Col |
| 1301/6 | 19.63 |  | Ler |  | Ler |  | Col |  | Col |  | Col |  | Col |  | Col |
| 1262/21 | 21.63 |  | Ler |  | Ler |  | Ler |  | Col |  | Col |  | Col |  | Col |
| 1254/4 | 25.29 |  | Ler |  | Ler |  | Ler |  | Col |  | Col |  | Col |  | Col |
| 1267/5 | 24.09 |  | Ler |  | Ler |  | Ler |  | Col |  | Col |  | Col |  | Col |
| 1267/6 | 30.65 |  | Ler |  | Ler |  | Ler |  | Col |  | Col |  | Col |  | Col |
| 1340/6 | 23.77 |  | Ler |  | Ler |  | Ler |  | Col |  | Col |  | Col |  | Col |
| 1369/10 | 32.11 |  | Ler |  | Ler |  | Ler |  | Ler |  | Ler |  | Col |  | Col |
| 1369/12 | 33.62 |  | Ler |  | Ler |  | Ler |  | Ler |  | Ler |  | Col |  | Col |
| 1369/24 | 49.3 |  | Ler |  | Ler |  | Ler |  | Ler |  | Ler |  | Col |  | Col |
| 1369/34 | 49.55 |  | Ler |  | Ler |  | Ler |  | Ler |  | Ler |  | Col |  | Col |

a Col is shown in green, Ler is purple and the region of unknown genotype containing the recombination is in grey.

b Physical position along chromosome 2

c Percentage of seeds germinated as described in Materials and Methods section
