# Supplementary material for: The Maternally Expressed WRKY Transcription Factor TTG2 Controls Lethality in Interploidy Crosses of Arabidopsis
Source: PLoS Biol. 2008 Dec 9;6(12):e308. doi: 10.1371/journal.pbio.0060308 (PMC2596861; doi:10.1371/journal.pbio.0060308)
Supplement: Supplementary file 4 [file pbio.0060308.st003.doc]

**Supplemental Table 3.** Summary of significant pair-wise marker interactions affecting interploidy lethality in Ler x Col RIL crossed to tetraploid Col.

|  |  |  |  |  | Likelihood Ratios | | | |
| --- | --- | --- | --- | --- | --- | --- | --- | --- |
| Pair of QTL | *DSL1* Marker | | *EOP* marker | | Whole model | *DSL1* marker | *EOP* marker | Interaction term |
| *DSL1* x *EOP1* | Chr2 | g17288 | Chr3 | ATHCHIB | 31.0 | 21.3 | 4 | 6.9 |
|  |  | g17288 |  | mi172 | 31.0 | 21.3 | 1.5 | 7.8 |
|  |  | nga168 |  | mi172 | 26.7 | 15.7 | 1.5 | 8.5 |
|  |  | BIO2b |  | mi172 | 26.5 | 15.2 | 1.5 | 9.1 |
|  |  | nga168 |  | mi467 | 27.9 | 15.7 | 1.6 | 9.9 |
|  |  | BIO2b |  | mi467 | 27.7 | 15.2 | 1.6 | 10.5 |
|  |  | nga168 |  | mi357 | 30.2 | 15.7 | 2.5 | 11.4 |
|  |  | BIO2b |  | mi357 | 30.0 | 15.2 | 2.5 | 12 |
|  |  | m336 |  | mi357 | 27.9 | 9.4 | 2.5 | 16.1 |
|  |  | g17288 |  | mi403 | 30.0 | 21.3 | 2.6 | 7.0 |
|  |  | nga168 |  | mi403 | 31.5 | 15.7 | 2.6 | 12.6 |
|  |  | BIO2b |  | mi403 | 31.5 | 15.2 | 2.6 | 13.3 |
|  |  | m336 |  | mi403 | 29.0 | 9.4 | 2.6 | 16.8 |
|  |  | mi277 |  | m583 | 27.4 | 18.6 | 2.1 | 7.5 |
|  |  | nga168 |  | m583 | 30.1 | 15.7 | 2.1 | 11.8 |
|  |  | BIO2b |  | m583 | 30.0 | 15.2 | 2.1 | 12.5 |
|  |  | m336 |  | m583 | 27.3 | 9.4 | 2.1 | 15.5 |
|  |  | g17288 |  | g13951 | 29.5 | 21.3 | 1.3 | 6.9 |
|  |  | nga168 |  | g13951 | 29.4 | 15.7 | 1.3 | 11.9 |
|  |  | BIO2b |  | g13951 | 30.4 | 15.2 | 1.3 | 13.4 |
|  |  | m336 |  | g13951 | 28.2 | 9.4 | 1.3 | 17.4 |
|  |  | nga168 |  | nga162 | 28.0 | 15.7 | 4.2 | 8.4 |
|  |  | BIO2b |  | nga162 | 27.7 | 15.2 | 4.2 | 8.9 |
|  |  |  |  |  |  |  |  |  |
| *DSL1* x *EOP2* | Chr2 | mi277 | Chr5 | nga139 | 29.5 | 18.6 | 0.9 | 9.7 |
|  |  | mi277 |  | mi174 | 26.8 | 18.6 | 1.8 | 7.2 |
|  |  | g17288 |  | nga139 | 30.9 | 21.3 | 0.9 | 8.5 |

The fit of a two locus model was tested by regression using the epistasis options within MapManagerQTX (Manly et al 2001). Significance thresholds were determined empirically by selecting the best marker-pair after each of 1000 permutations of the data. We chose an LR of 26.5 for the whole model as the significance threshold for this experiment, corresponding to an estimated p<0.05.

All marker pairs with LR exceeding this threshold are reported in Supplemental Table 3. In addition the LR corresponding to each individual marker and their interaction term, indicative of the epistasis, are presented.
