# Supplementary material for: The Maternally Expressed WRKY Transcription Factor TTG2 Controls Lethality in Interploidy Crosses of Arabidopsis
Source: PLoS Biol. 2008 Dec 9;6(12):e308. doi: 10.1371/journal.pbio.0060308 (PMC2596861; doi:10.1371/journal.pbio.0060308)
Supplement: Supplementary file 5 [file pbio.0060308.st004.doc]

**Supplemental Table 4.** Main and interaction effects for %Live seed in Col x Ler RIL.

| QTL | Marker | QTL | Marker | Effect | F-ratio | p-value |
| --- | --- | --- | --- | --- | --- | --- |
| DSL | g17288 |  |  | -0.10 | 34.8121 | <0.0001 |
| EOP1 | ATHCHIB |  |  | -0.01 | 0.0878 | 0.7679 |
| EOP2 | nga139 |  |  | -0.04 | 5.5776 | 0.0209 |
| DSL | g17288 | EOP1 | ATHCHIB | ^a^ | 9.4051 | 0.0030 |
| DSL | g17288 | EOP2 | nga139 | ^a^ | 8.5162 | 0.0047 |
| Model | R^2^=0.40 | F-ratio 9.6; df 5,72; p-value <0.0001 | | | | |

To better estimate the role of the epistatic interactions on the phenotypic distribution in the RIL and further rule out spurious false-positive detection of epistasis DSL and both epistatic interactions were analyzed simultaneously by multiple regression. For this the best marker showing interaction with both EOP1 and EOP2 was chosen to model DSL1, g17288, and the markers ATHCHIB and nga139 were used to represent EOP1 and EOP2, respectively. A regression model including each marker’s main effect, as well as the pair-wise interactions between DSL1 and EOP1 and DSL1 and EOP2 was tested for fit the seed fertility data from the RIL. The findings are summarized in Supplemental Table 4. The QTL name and marker(s) are presented in the first two columns. In the last two rows, a pair of marker/QTL are listed for each of the two epistatic interactions. In the Effect column, the mean difference in %plump affected by of being a homozygous at the listed marker is presented with the effect of the Col allele presented. Significance for the association between maker and phenotype is then presented as both the F-statistic for each regression coefficient and as the p-value for that regression coefficient. Both interactions terms, and the main effect at DSL1 and EOP2 were significant.

The distribution of %live seed within the RIL population was not normal (See Supplemental Figure 1; analysis not shown). Non-normality in the data can cause spurious detection of epistasis. Box-Cox transformation of the data to improve normality (not shown) actually increased the significance of all terms except the main effect of EOP1. This indicates that the epistasis detected is not a result of non-normality in the data set. The residuals following multiple regression using a single marker corresponding to the *DSL1*, *EOP1* and *EOP2* regions were normally distributed (analysis not shown) consistent with an underlying multivariate normal distribution.
