# Supplementary material for: The Maternally Expressed WRKY Transcription Factor TTG2 Controls Lethality in Interploidy Crosses of Arabidopsis
Source: PLoS Biol. 2008 Dec 9;6(12):e308. doi: 10.1371/journal.pbio.0060308 (PMC2596861; doi:10.1371/journal.pbio.0060308)
Supplement: Supplementary file 6 [file pbio.0060308.st005.doc]

**Supplemental Table 5.** *fis2-8* did not modulate viability in crosses to tetraploid Col.

| Seed parent | Plump seeds (N) | Shriveled seeds (N) | % Plump Seeds |
| --- | --- | --- | --- |
| *FIS2/FIS2* | 10 | 51 | 16.4 |
| *fis2-8/FIS2* | 12 | 130 | 8.5 |

The *fis2-8* allele was backcrossed into the Col background for 9 generations. The *fis2-8* mutation results in 50% seed death for a heterozygous mother due to female gametophyte-dependent lethality. If *FIS2* encodes the *DSL1* QTL, a maternal sporophytic effects of the *fis2-8* allele on interploidy crossing success should result in a change in the success rate of hybridization between wild-type haploid ovules and diploid pollen using *fis2-8/FIS2* heterozygotes as opposed to homozygous wildtypes. The null hypothesis, of no effect on hybridization efficiency, would result in half the number of surviving progeny following crosses of *fis2-8/FIS2* to tetraploid Col as in crosses of wild-type diploid Col to tetraploid Col. The observed frequency of live seed does not deviate from the null hypothesis (Chi-square p-value > 0.05.
