# Supplementary material for: The Maternally Expressed WRKY Transcription Factor TTG2 Controls Lethality in Interploidy Crosses of Arabidopsis
Source: PLoS Biol. 2008 Dec 9;6(12):e308. doi: 10.1371/journal.pbio.0060308 (PMC2596861; doi:10.1371/journal.pbio.0060308)
Supplement: Supplementary file 7 [file pbio.0060308.st006.doc]

**Supplemental Table 6.** TTG2 modulates mature seed size in Ler and Col genetic backgrounds.

|  | seed length mm^a^ | seed width mm^a^ | seed weight mg |
| --- | --- | --- | --- |
| Col-0 | 415.20 ± 4.16 (n=50) | 263.58 ± 2.70 (n=50) | 19.6 (n=400) |
| *ttg2-3* | 393.38 ± 3.48 (n=50) | 267.70 ± 2.12 (n=50) | 18.8 (n=423) |
| L*er* | 410.72 ± 5.07 (n=50) | 271.26 ± 2.81 (n=50) | 16.9 (n=240) |
| *ttg2-1* | 342.84 ± 4.20 (n=50) | 247.82 ± 2.66 (n=50) | 13.6 (n=226) |

^a^± standard error of the mean
