# Supplementary material for: The Maternally Expressed WRKY Transcription Factor TTG2 Controls Lethality in Interploidy Crosses of Arabidopsis
Source: PLoS Biol. 2008 Dec 9;6(12):e308. doi: 10.1371/journal.pbio.0060308 (PMC2596861; doi:10.1371/journal.pbio.0060308)
Supplement: Supplementary file 8 [file pbio.0060308.st007.doc]

**Supplemental Table 7.** *TTG2* modulates seed viability in crosses of diploid mutants to tetraploid Col.

| Seed parent | Plump seeds (N) | Shriveled seeds (N) | Plump seeds (%) | P-value for WT vs. mutant t-test |
| --- | --- | --- | --- | --- |
| Wildtype Col | 27 | 171 | 13.6 |  |
| *ttg2-3* | 71 | 40 | 64.0 | <0.0001 |
|  |  |  |  |  |
| Wildtype Ler | 149 | 140 | 51.6 |  |
| *ttg2-1* | 107 | 29 | 78.7 | 0.01 |

The quantitative data corresponding the investigation of a role for *TTG2* in controlling interploidy cross fertility (Figure 6) is summarized in supplemental table 4. Two loss of function alleles of *TTG2*, one in the Col background and one in the Ler background, were tested for their effects on seed survival. Wild-type siblings of *ttg2-3* derived from the same T5 pool were used as controls.
