# Supplementary material for: The Maternally Expressed WRKY Transcription Factor TTG2 Controls Lethality in Interploidy Crosses of Arabidopsis
Source: PLoS Biol. 2008 Dec 9;6(12):e308. doi: 10.1371/journal.pbio.0060308 (PMC2596861; doi:10.1371/journal.pbio.0060308)
Supplement: Supplementary file 9 [file pbio.0060308.st008.doc]

**Table S8**. Oligonucleotide primers used in this study

| Primer name | Primer sequences |  | Chr. | Positionin cM | Position in bp | PCR program | Marker Type (RE) a | Size in bp-Ler | Size in bp-Col |
| --- | --- | --- | --- | --- | --- | --- | --- | --- | --- |
| nga361 | ACATATCAATATATTAAAGTAGC | AAAGAGATGAGAATTTGGAC | 2 | 63.0 | 13229491 | ssr50 | SSLP | 120 | 114 |
| FIS2 | TGCTGCATGTGAATGATGAA | GACCCTTAAGACGCTCCAAT | 2 |  | 15002122 | td62-52 | SSLP | 967 | 787 |
| ve017 | GAGCAATCCAGTAGAGGATA | CTTGAAGCTTAAATCTCAGC | 2 | 69.14 | 15452540 | td62-52 | CAPS(PstI) | 357 | 214,143 |
| nga168 | GAGGACATGTATAGGAGCCTCG | TCGTCTACTGCACTGCCG | 2 | 73.77 | 16298919 | ssr50 | SSLP | 135 | 151 |
| nga172 | CATCCGAATGCCATTGTTC | AGCTGCTTCCTTATAGCGTCC | 3 | 6.91 | 786303 | ssr50 | SSLP | 136 | 162 |
| nga162 | CTCTGTCACTCTTTTCCTCTGG | CATGCAATTTGCATCTGAGG | 3 | 20.56 | 4608284 | ssr50 | SSLP | 89 | 107 |
| nga1107 | CGACGAATCGACAGAATTAGG | GCGAAAAAACAAAAAAATCCA | 4 | 104.73 | 1809613 | ssr50 | SSLP | 140 | 150 |
| TTG2 7bp | TTCAACTAAAACCAAACGACACC | TGTGATGGATACACGTGTCAGTA | 2 |  |  | ssr50 | Indel |  |  |
| TTG2 PERL0390427 | gatccatccaacgttcccta | ttcaccatcattcacctcca | 2 |  |  | ssr50 | SNP |  |  |
